# 130 Promoting health enhancing physical activity through social prescribing in Wales: A delivery and recommendations framework for nature-based wellbeing support programmes

**DOI:** 10.1093/eurpub/ckae114.104

**Published:** 2024-09-26

**Authors:** Paul Sellars, Diane Crone, Jenny Mercer, Debbie Clayton

**Affiliations:** Centre for Health, Activity and Wellbeing Research (CAWR), Cardiff Metropolitan University, Cardiff, Wales; Centre for Health, Activity and Wellbeing Research (CAWR), Cardiff Metropolitan University, Cardiff, Wales; Centre for Health, Activity and Wellbeing Research (CAWR), Cardiff Metropolitan University, Cardiff, Wales; Centre for Health, Activity and Wellbeing Research (CAWR), Cardiff Metropolitan University, Cardiff, Wales

## Abstract

**Purpose:**

In 2024 the Welsh Government published the National Framework for Social Prescribing, which sets out their plan for social prescription throughout Wales. Generally, social prescribing is a means of connecting people to non-clinical services in their community to improve health and wellbeing (WHO, 2022). ‘Wild Skills, Wild Spaces’ (WSWS) is an ecotherapy based social prescribing project run by Montgomeryshire Wildlife Trust. WSWS provides nature-based physical activity aimed at improving the health and wellbeing of people with mental health problems, as well as supporting the management and conservation of the sites where it is delivered. The Centre for Health, Activity and Wellbeing Research (CAWR), commissioned by the Montgomeryshire Wildlife Trust, conducted a two-year mixed-methods evaluation resulting in findings that confirmed the role WSWS has for both health and wellbeing benefits through being active. Informed by the findings and in alignment with the Welsh National Framework for Social Prescribing, the current project sought to develop a transferable delivery and recommendations framework for programmes in nature, aimed at enhancing physical, mental, and social wellbeing for people with mental health problems.

**Methods:**

The ‘Delivery and Recommendations Framework for Nature-based Wellbeing Support Programmes’ was developed through a collaborative and iterative process incorporating four aspects: (i) WSWS evaluation findings, (ii) relevant peer reviewed journal articles, (iii) collaborative discussions with the WSWS staff, and (iv) the context of the Welsh National Framework for Social Prescribing.

**Results:**

Following the iterative process, a delivery and recommendations framework for nature-based wellbeing support programmes was developed, encompassing a diagram and six tables. The diagram reflects the Welsh National Framework for Social Prescribing and locates where nature-based activities fit within the framework. The six tables include recommendations/guidelines for nature-based conservation programmes pertaining to:

• Social prescribing services

• People

• Activities

• Facilities

• Setting/environment

• Continued engagement and sustainability.

**Conclusion:**

Overall, the delivery and recommendations framework provide recommendations/guidelines for people designing and delivering programmes in nature, aimed at promoting health enhancing physical activity, and mental and social wellbeing. The recommendations/guidelines encompass six delivery areas, and are transferable to other setting across Europe.

**Support:**

WSWS was funded by the Welsh Government.

